# Cook County Hospital Case of Tetanus Treated by Nerve Stretching; Recovery

**Published:** 1882-10

**Authors:** 


### Hospital Reports.

### ARTICLE VI.

### COOK COUNTY HOSPITAL, CHICAGO.

### CASE OF TETANUS, TREATED BY NERVE-STRETCHING. RECOVERY.

The following is a condensation of the notes taken of the case by the house surgeon, Dr. Mary E. Bates.

Frank Periolat, æt. nine years, had his right foot and leg run over by a street car, June 16, 1882. He was admitted to the hospital the same day. He gave evidence of previous good health, and the shock following his injury was not severe. Examination revealed extensive laceration of the soft parts, a contused wound of the foot, and a compound fracture of the leg, necessitating amputation, which was performed the same day by Dr. Baxter, attending surgeon. The patient having been anæsthetized, the leg was amputated between the upper and middle third, posterior and anterior flaps being brought together, after ligating the vessels, with catgut. A rubber drainage tube was left in the wound, and the flaps were united with silk sutures. The pulse beat 106 a minute, and the temperature of the patient was 92° Fahrenheit. He made a slow but full reaction.

In the forenoon of June 17, patient's pulse ran up to 128, and his temperature to 104.6. He was restless, but did not manifest any pain. He received half a drachm of paregoric twice. He took some breakfast, was given a sponge bath, and took three grains of quinine sulphate three times a day. That day his bowels moved. In the afternoon his pulse was 132, and his temperature 103.4. The next day the pulse returned to 110, and temperature 100.8.

June 19, the dressings were renewed. There was moderate suppuration. A smaller drainage tube was substituted. The pulse and temperature remained about stationary till June 30. The sutures and drainage tube were removed June 23, and the dressings changed. There was a slight amount of suppuration. June 28, only a superficial wound remained.

July 1, patient complained of pain and stiffness in the back of his neck, with inability to open the jaws more than one-fourth inch. This condition the patient first noticed the morning of the previous day.

July 1, patient received by hypodermic injection one sixty-fourth of a grain of physostigma and salicylic extract at six P. M., again at 8:30, and at 9:30. At midnight he had spasms of the glottis, and symptoms of opisthotonos.

July 2, one A. M., he received one sixty-fourth gr. of physostigma in the same manner as before, and took per orem 24 grains of potassium bromide and 15 grains of chloral hydrate, in solution. The pupils were moderately dilated, and patient was quiet. At 2:25 A. M. his pulse was 90, and the pupils were well contracted. Patient asleep. The injection was repeated at 3:15, and at 4 A. M. the jaw was slightly relaxed. 5:30, pupils dilated; injection repeated; pulse 104 to 99.5. 8 A. M. another injection, and 25 grs. of bromide with 15 grs. of chloral per orem. Patient could open the jaw three-eighths inch. The stump of the amputated limb was re-dressed. It looked well. Physostigma was given by hypodermic injection at 11, 12, and at 1 P. M. The jaws opened one-fourth inch. Injections were repeated at one and a half hours interval. Pulse was 122; temperature 100.6. Very little pain was complained of in the back of the neck and head. The risus sardonicus, which had been present some time, now disappeared; spasms ceased. One twenty-fifth gr. physostigma was given every hour. About midnight slight spasms occurred.

July 3, injections continued, except that a few doses were taken per orem instead of hypodermically. The pupils remained dilated; the pulse was 106, and the temperature 100. The risus sardonicus, a drawing downward of the corners of the mouth, recurred about 10 A. M. Injections were given nearly every hour.

July 4. Pulse 132; temperature 102.1 The hypodermic injections of Calabar bean were given every one and a half hour. Patient had convulsions at 8 and 9 A. M., at noon, and at 2:20 and 4 P. M.

July 5, 11 A. M., the patient was etherized, the stump was opened and scraped, one-eighth inch of a nerve was removed, but no spicula of bone nor any other source of irritation was found. The granulations were flabby. A drainage tube was re-introduced into the wound, and some sutures applied, and stump dressed antiseptically. Dr. E. W. Lee made an incision below the gluteal region, two inches in length, and the sciatic nerve was stretched. The anterior crural nerve was also exposed and stretched. Both incisions were antiseptically closed after drainage tube had been introduced. The pulse was rapid, and not very strong. Half a drachm of brandy was given hypodermically. The patient slept all the afternoon under the influence of the ether. In the afternoon his pulse was 121, and temperature 101. At 8 P. M. bromide of potassium with chloral were given, together with one-sixteenth gr. of morphia, which latter was repeated at 9 P. M., and the two former at 10:30. The pupils were contracted, and the pulse was 132.

July 6, 12:30 A. M., the patient had convulsions, which were somewhat controlled by chloroform given by inhalation. At 1 A. M., the bromide and chloral were repeated, also at 2, 3, and 4 A. M. Pupils contracted. During the above convulsions, the tongue was protruded and livid, and there was opisthotonos and tonic spasms of the voluntary muscles ; trismus ; rapid and stertorous breathing ; threatened asphyxia. The chloroform administered relaxed the muscles. The convulsions lasted ten minutes. The following anæsthesia was complete. At 4 A. M. another severe convulsion set in, which was controlled in the manner of the previous one. The chloroform relaxed the pupils. At 7 A. M. one-eighth gr. of morphine was given hypodermically. The pupils contracted, and there was a tonic contraction of the muscles of the neck. 8:30, bromide, chloral, and morphine were given, and the two first were repeated hourly for nine hours. 7 P. M., convulsions, less severe than and treated as the preceding ones.

July 7, hypodermics of morphia were given hourly for six hours. Pulse 118; temperature 99.9. There was no spasm that night. Bromide and chloral were administered at 9 and 10 A. M., and the patient could open his mouth an inch. Morphine was given alternately with chloral and bromide every hour or two till midnight, when there was another attempt at spasm, and stertorous breathing.

July 8, 4 A. M., some signs of spasm; 8 A. M., severe convulsion, controlled by chloroform; former treatment continued. That day, the drainage tubes were rèmoved from the incisions over the sciatic and crural nerves. The stump of the amputated limb was dressed, and a sanious material was discharged. After irrigation, antiseptic dressings were re-applied, and the treatment continued. At noon the patient drank plenty of milk and coffee. The bromide and chloral mixture was now given every two hours, alternately with hypodermic injections of morphine. Lavements were given to move the bowels, which, however, had been regular till that day, and they moved freely the next morning. The same treatment was continued till July 10, after which the two mixtures were given every three hours.

July 10, pulse 126; temperature 100.5. July 11, there was yet some trismus, but the patient could open his mouth wider. There was a good deal of salivation. July 15, the pulse was 110, and the temperature 99; he was feeling better. Patient subsequently got well.

---

ROCKFORD, ILL., AUG. 15, 1882.

MESSRS EDITORS:—If a couple of feet of small rubber tubing be attached to an ordinary "finger-stall," such as is found in most drug stores, and its tip, sewed to an elastic band, be pushed into the ear, the stalled finger will convey sounds, in percussion, where nicety is requisite, far better than the ordinary way. It is to percussion, what the stethoscope is to auscultation. An apparatus (cost 28 cents) which I devised yesterday, worked nicely. Should this be worth publishing, use it.

Yours truly, E. C. HUSE, M.D.

